# Le syndrome d'Othello: un cas observé à Ouagadougou

**DOI:** 10.11604/pamj.2013.16.6.2556

**Published:** 2013-09-05

**Authors:** Bawindsongré Jean Kaboré, Christian Napon

**Affiliations:** 1 Service de neurologie, CHU Yalgado Ouedraogo, BP 7022, Ouagadougou 03, Burkina Faso

**Keywords:** syndrome d'Othello, délire de jalousie, AVC, Ouagadougou, Othello syndrome, delusional jealousy, CVA, Ouagadougou

## Abstract

Les auteurs rapportent le premier cas de délire de jalousie encore décrit sous l'acronyme de syndrome d'Othello, à Ouagadougou. Il s'est agit d'un patient qui au décours d'un accident vasculaire cérébral ischémique constitué, a développé un délire de jalousie. Une revue de la littérature permet de comprendre que l'affection, rarement rapportée est de plus en plus décrite au décours de maladies neurologiques aigues ou chroniques, et que le lobe frontal joue vraisemblablement un rôle majeur.

## Introduction

Le syndrome d'Othello ou encore délire de jalousie réalise un délire passionnel dont le thème porte sur l'infidélité supposée du partenaire. Il est rarement rapporté dans la littérature. Il s'agit d'une affection psychiatrique décrite dans le contexte de psychose organique (démence, alcoolisme) ou fonctionnelle (schizophrénie, paranoïa). Récemment, quelques cas ont été décrits au cours de la maladie de Parkinson [1]. Nous rapportons le premier cas observé dans le service de Neurologie du CHU de Ouagadougou.

## Patient et observation

J S, sexe masculin est âgé de 46 ans; Il est évangéliste, consomme modérément l'alcool; il est marié, sans difficultés conjugales déclarées; son épouse est sans travail rémunéré, femme au foyer. J S est hypertendu connu, mal suivi depuis quelques mois; il est obèse, avec un indice de masse corporelle à 32 kg/m2, reçu dans le service de neurologie pour déficit moteur brutal de l'hémicorps droit avec aphasie; le bilan clinique avait conclu à un AVC et la tomodensitométrie cérébrale avait objectivé une hypodensité du thalamus et du bras postérieur de la capsule interne gauches. Les explorations biologiques avaient objectivé par ailleurs une hyper uricémie; le reste du bilan était normal, notamment le MMS >23. Le NIHSS n’était pas de pratique courante dans le service. La prise en charge de J S a consisté en un traitement de l'hypertension artérielle par nifédipine 20 mg/jour, un traitement antiagrégant plaquettaire par acide salicylique 250 mg/jour et une rééducation motrice. L'hyperuricémie est traitée par allopurinol 200 mg/jour. La récupération motrice a été médiocre, la récupération du langage moyenne; progressivement avec la récupération clinique s'est installé un syndrome frontal avec une humeur expansive, mais surtout une agressivité sélective envers son épouse que le patient accusait d'infidélité au profit d'un homme non identifié. Les sorties de l’épouse auraient été des occasions pour rencontrer cet individu qui aurait envisagé donc de l'empoisonner. Depuis lors, J S a refusé les mets préparés par sa conjointe mais a observé tout de même le traitement qui avait été prescrit, et administré par son épouse. Malgré le traitement neuroleptique proposé à base d'halopéridol, le délire est resté persistant sur le même thème, pendant plus de deux ans.

## Discussion

Cette observation réalise un tableau de délire de jalousie contemporain à un accident vasculaire cérébral ischémique, chez un patient présentant des facteurs de risque vasculaires: HTA, obésité, hyperuricémie. Le délire a émergé dès la récupération du déficit moteur, mais surtout de l'aphasie motrice; il était associé à un syndrome frontal avec une humeur expansive et une agressivité, certes sélective. Le thème du délire nous a d'emblée orienté vers les délires paranoïdes. Dans ce groupe on décrit le délire de grandeur, le délire érotomaniaque, le délire de persécution, le délire de jalousie, le délire mixte [2]. Les délires paranoïdes sont surtout décrits en psychiatrie mais la littérature rapporte de plus en plus de cas, en rapport avec des affections somatiques neurologiques telles les épilepsies, les traumatismes crânio-encéphaliques, les affections vasculaires et les affections dégénératives; parmi ces dernières, on cite les démences, la maladie de Parkinson [3]

Le syndrome d'Othello rappelle le personnage d'opéra de Shakespeare, un maure habitant de Venise; Othello suspectant sa femme d'infidélité suite à une machination de son compagnon d'armes, la tua mais se rendit compte plus tard de son erreur.

L'observation que nous rapportons s'illustre par l’émergence de la symptomatologie psychiatrique par rapport à l’évolution de l'AVC: c'est à la faveur de la récupération motrice et phasique que le délire a émergé. Le délire est peut-être mixte, quand on considère le thème de l'empoisonnement souvent évoqué par le patient. Les conséquences cognitives, psychologiques, et comportementales des affections neurologiques dépendent de plusieurs paramètres, qui entre autres sont: le mode évolutif de l'affection, le siège de la lésion, le système de neurotransmetteur impliqué, le facteur individuel (sexe, âge, éducation, croyance...): les affections aigües auront comme conséquences des états confusionnels ou des délires, alors que les affections chroniques retentiront sur l'attention et la cognition [3].

Le diagnostic du siège de la lésion nous a posé quelques difficultés pour le rapporter au tableau clinique; en effet, l'hypodensité notée à la tomodensitométrie intéresse le thalamus et le bras postérieur de la capsule interne gauches, alors que dans la littérature les zones du cerveau impliquées dans la survenue de syndrome d'Othello sont les régions frontales des hémisphères droit et/ou gauche, les avis sont encore partagés [1, 4, 5]. Nous pensons que d'autres lésions non évidentes sur la TDM sont responsables de la symptomatologie chez J S (par exemple, les lésions corticales responsables de l'aphasie ne sont pas visualisées sur cette tomodensitométrie).

## Conclusion

Les mécanismes physiopathologiques du syndrome d'Othello et des autres délires dans les affections organiques restent mal connus, même si certaines études de laboratoires mettent en évidence le rôle activateur du cortex frontal. Le traitement reste décevant avec les neuroleptiques classiques [1]; les neuroleptiques atypiques auraient de meilleurs résultats [2]. Du fait de sa possible survenue au décours des AVC, le syndrome d'Othello doit être connu.
